# Using Digital RNA Counting and Flow Cytometry to Compare mRNA with Protein Expression in Acute Leukemias

**DOI:** 10.1371/journal.pone.0049010

**Published:** 2012-11-09

**Authors:** Paula Fernandez, Max Solenthaler, Olivier Spertini, Stephane Quarroz, Alicia Rovo, Pierre-Yves Lovey, Leda Leoncini, Sylvie Ruault-Jungblut, Mathilde D’Asaro, Olivier Schaad, Mylène Docquier, Patrick Descombes, Thomas Matthes, Swiss Cytometry Society

**Affiliations:** 1 Kantonsspital Aarau, Aarau, Switzerland; 2 University Clinic for Hematology and Central Hematology Laboratory, Inselspital Bern and University of Bern, Bern, Switzerland; 3 Hematology Service, CHUV, University Hospital Lausanne, Lausanne, Switzerland; 4 Hematology Department, University Hospital Basel, Basel, Switzerland; 5 Hematology Service, Institut Central des Hôpitaux Valaisans, Sion, Switzerland; 6 Hematology Service, Istituto Oncologico della Svizzera Italiana, Bellinzona, Switzerland; 7 Hematology Service, University Hospital Geneva, Geneva, Switzerland; 8 Genomics Platform, CMU, University Medical Center, Geneva, Switzerland; Cincinnati Children's Hospital Medical Center, United States of America

## Abstract

**Background:**

The diagnosis of malignant hematologic diseases has become increasingly complex during the last decade. It is based on the interpretation of results from different laboratory analyses, which range from microscopy to gene expression profiling. Recently, a method for the analysis of RNA phenotypes has been developed, the nCounter technology (Nanostring® Technologies), which allows for simultaneous quantification of hundreds of RNA molecules in biological samples. We evaluated this technique in a Swiss multi-center study on eighty-six samples from acute leukemia patients.

**Methods:**

mRNA and protein profiles were established for normal peripheral blood and bone marrow samples. Signal intensities of the various tested antigens with surface expression were similar to those found in previously performed Affymetrix microarray analyses. Acute leukemia samples were analyzed for a set of twenty-two validated antigens and the Pearson Correlation Coefficient for nCounter and flow cytometry results was calculated.

**Results:**

Highly significant values between 0.40 and 0.97 were found for the twenty-two antigens tested. A second correlation analysis performed on a per sample basis resulted in concordant results between flow cytometry and nCounter in 44–100% of the antigens tested (mean = 76%), depending on the number of blasts present in a sample, the homogeneity of the blast population, and the type of leukemia (AML or ALL).

**Conclusions:**

The nCounter technology allows for fast and easy depiction of a mRNA profile from hematologic samples. This technology has the potential to become a valuable tool for the diagnosis of acute leukemias, in addition to multi-color flow cytometry.

## Introduction

Diagnosis of leukemia and lymphoma is in part based on the recognition of surface antigens expressed by the tumor cells. So far, over 360 hematopoietic membrane markers have been classified as “clusters of differentiation” (CD), and the study of various combinations of these antigens by flow cytometry is used in the work-up of patients with a suspected hematologic neoplasia. In practice, routine flow cytometry laboratories use specific antibodies against only 30–50 of the known antigens for the differential diagnosis of leukemias and lymphomas. The extent of the panels used depends on the laboratories expertise, its resources and the costs of these analyses, which become increasingly expensive and time-consuming the more antibodies are used.

Several other methods are currently being developed, which, like flow cytometry, allow the study of a set of simultaneously expressed protein antigens. Mass cytometry, a new technology based on atomic mass spectrometry, offers the possibility to analyze literally hundreds of different surface antigens in one single assay [1]. Proof of principle of this technology in leukemia diagnosis has already been reported [2]. Another approach uses antibody microarrays based on a solid phase cell-capture technique [3], [4]. Belov L et al. reported the analysis of the simultaneous expression of eighty-two antigens in 733 patients with a variety of leukemias and lymphomas, achieving a high degree of diagnostic consensus between established criteria and the results of an antibody microarray (93.9% for peripheral blood and 97.6% for bone marrow aspirates) [3]. Although this approach potentially offers the possibility to analyze in parallel several hundreds of antigens, the drawbacks are the limitation to surface proteins and the rather low sensitivity, since more than 20% of blasts have to be present in a sample in order to recognize its malignant signature. Other methods of protein phenotyping have, to our knowledge, not yet reached the clinical practice.

An alternative approach for the analysis of the biological state of a cell lies in the analysis of its mRNA profile. Indeed, as protein profiling, methods of mRNA profiling (gene expression profiling, GEP) using microarray technology have been developed in the last decade and have been already extensively validated in the field of lymphoma and leukemia diagnosis [5], [6]. Recently, the company Nanostring has developed a new high-throughput RNA expression profiling system (nCounter), which allows the direct digital readout of mRNA molecules and their relative abundance using small amounts of total RNA (100 ng), without requiring cDNA synthesis or enzymatic reactions [7]. Several groups have already compared the nCounter method, the Affymetrix platform, and standard quantitative RT-PCR, and observed high correlation coefficients above 0.9 for values obtained by any of the three methods [7], [8], [9], [10].

So far, only a few studies have attempted to correlate the findings of both types of approaches, of protein and of mRNA expression analysis. Kern et al. compared data from flow cytometry and mRNA-microarrays on thirty-six antigens from 814 patient samples. They found heterogeneous correlation coefficients between the two techniques, ranging from rather low to high correlation coefficients for certain antigens (e.g. 0.17 for CD19 and 0.80 for CD34, respectively) [11].

We performed the present study with the aim to validate the nCounter technology for the diagnosis of acute leukemias, by comparing mRNA profiles obtained by this methodology to protein profiles obtained by flow cytometry. We studied eighty-six samples from acute leukemia patients in a multi-center study and found high correlation for most antigens relevant in routine flow cytometric analyses (corr. coeff. from 0.40 for CD38 to 0.97 for CD133). We therefore propose this system as a new, extremely easy to use, rapid, and very informative method of analysis for the diagnosis of acute leukemias and other hematologic diseases.

## Results

### Validating the nCounter Probe Set

In order to investigate potential correlations between mRNA and protein expression in peripheral blood (PB) and bone marrow (BM) samples we choose a set of eighty-eight well-known antigens, most of them currently used for leukemia diagnosis in routine flow cytometry laboratories. Information about these antigens, in particular their expression profile in different cell populations present in PB and BM, was readily available from the literature and public databases (www.genecards.org; http://prow.nci.nih.gov).

**Figure 1:**
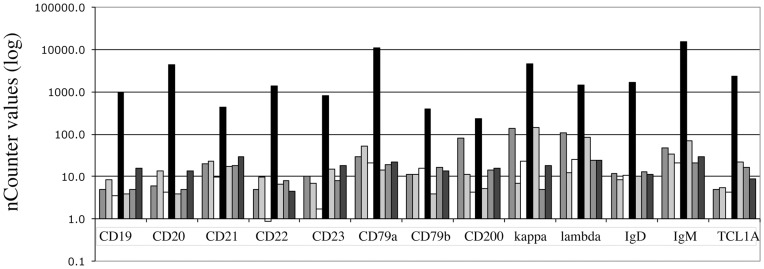
Analysis of mRNA expression of thirteen B cell specific antigens in seven different cell populations isolated from peripheral blood. For each antigen the columns from left to right correspond to CD4+ T-cells (dark gray), CD8+ T-cells (light gray), NK cells (white), CD19+ B-cells (black), CD14+ monocytes (light gray), CD33+ neutrophils (gray), and eosinophils (dark grey).

With custom-designed probes from Nanostring specific for these eighty-eight antigens we first analyzed seven different, highly purified, cell populations, sorted from peripheral blood (CD4+ T-cells, CD8+ T-cells, NK cells, CD19+ B-cells, CD14+ monocytes, CD33+ granulocytes, eosinophils). As example, probes for genes expressed solely by B cells, gave high signals in the sample with the purified B cell population and weak signals in the other six sorted populations containing only a few contaminating B cells (Figure 1). As expected, differences were in the 2- to 3-log range, depending on the probe and its specificity, the amount of mRNA/B cell, and the number of contaminating B cells in the other sorted cell samples. For other cell-type specific genes results were similar (data not shown).

### mRNA Expression Profiles in Normal Peripheral Blood and Normal Bone Marrow Samples

Cell lysates from eleven normal BM aspirates and from PB from five blood donors were analyzed with the nCounter. Means for signal intensities were calculated and ordered according to signal intensity and specific expression in the different cell subpopulations, e.g. T cells, B cells, etc. (Table S1). mRNA quantities for several cell-type specific genes varied between BM and PB, depending on the number of cells from a subpopulation present in one or the other of these two compartments, on their differentiation stage, and on the number of mRNA molecules/cell. For example, genes specific for blast cells were expressed essentially in normal BM, but not in PB samples (CD34 was increased 32-fold compared to PB, CD133 185-fold, and CD117 12-fold), whereas T-cell specific genes were found increased 9.5-fold ±2.9 in PB compared to BM, in accordance with the 2- to 3-fold higher number of T cells in PB.

### Comparison of mRNA and Protein Phenotypes

Twenty-two antigens with a particular relevance in flow cytometric diagnosis and used by most laboratories taking part in the study were chosen for a comparative analysis by flow cytometry and the nCounter technology.

This analysis was performed for seventy-three cases: normal bone marrow (11 samples), normal peripheral blood (5 samples), sorted cell populations (CD4+ T cells, CD8+ T cells, B cells, CD34+ blasts, monocytes, neutrophils, eosinophils and erythroid precursors), and acute leukemia patients (45 samples), CLL patients (2 samples), and CML patients (2 samples) (Table S2). This resulted in a total of 1606 comparisons.

**Figure 2:**
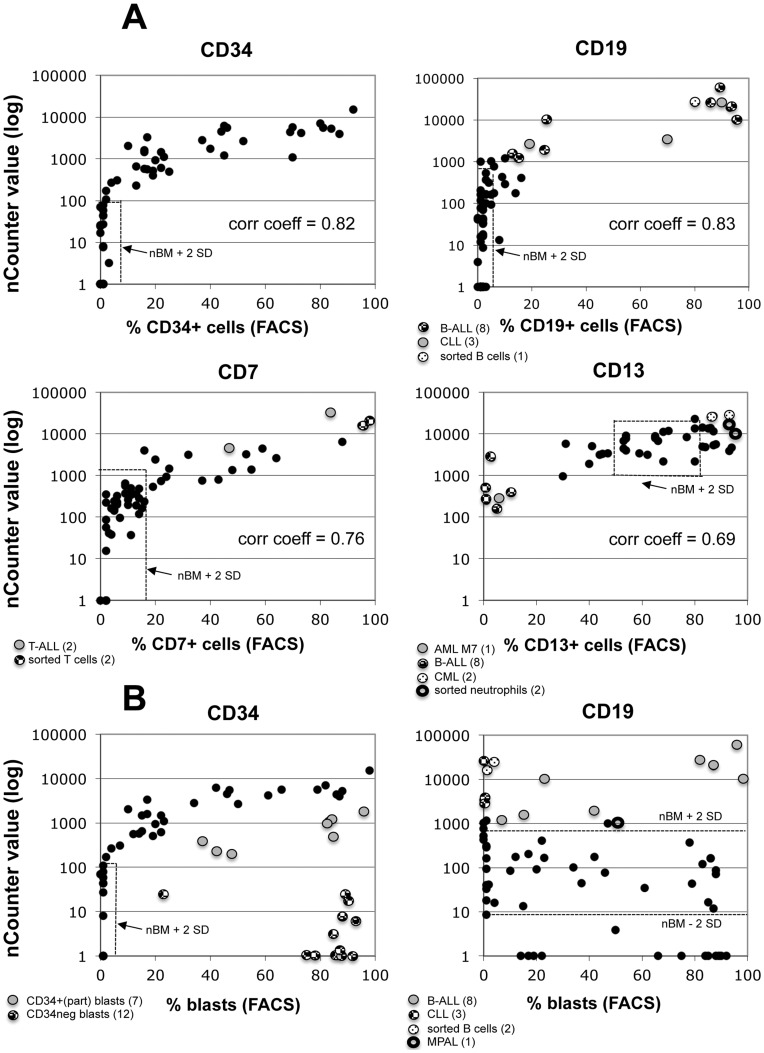
Correlation of flow cytometric and nCounter results. **A:** Correlation between results from the nCounter technology and flow cytometry for four typical antigens. Flow cytometric results are given as percentage of cells positive for a given antigen, and nCounter results as digital counts (after normalization and background subtraction). The number of depicted data points varies depending on the antigen tested and on the availability of both cytometric and nCounter values for a given sample. Only samples for which both values were measured are shown. Some samples, particularly informative for a particular antigen, were highlighted with dots in different shades and patterns. nBM, normal bone marrow; corr coeff, Pearson’s correlation coefficient. corr coeff >0.393 are statistically significant with p<0.01. **B:** Correlation between % of leukemic blasts as determined by flow cytometry, and nCounter values for two typical antigens (CD34, CD19).

The comparisons between signal intensities obtained by the nCounter analysis and percentages of positive cells as determined by flow cytometry using isotype controls resulted in highly significant correlations for all the genes analyzed. Figure 2A shows the results for four antigens: CD34, CD19, CD7, and CD13. Using Pearson’s correlation the coefficients ranged between 0.40 and 0.97 (Table 1).

**Table 1:** Correlation coefficient for flow cytometry (protein) and nCounter (mRNA) values from 22 antigens.

Antigens	corr coeff
CD133	0.97
CD3	0.94
CD20	0.89
TDT	0.89
CD19	0.83
CD34	0.82
CD10	0.81
CD56	0.80
CD11b	0.78
HLA-DR	0.76
CD7	0.76
CD117	0.75
CD16	0.74
CD4	0.68
CD13	0.61
CD36	0.57
CD14	0.56
MPO	0.53
CD123	0.53
CD33	0.49
CD15	0.41
CD38	0.40

Pearson’s correlation coefficient between nCounter signal intensity and percentage of positive cells measured by flow cytometry. Twenty-two different antigens were analyzed in seventy-three samples (11 normal bone marrow, 5 normal peripheral blood, 8 sorted cell populations, 45 acute leukemia, 2 CLL, and 2 CML).

corr coeff >0.302 are statistically significant with p<0.01.

In addition, MFI values obtained from flow cytometry analysis were compared with the results from the nCounter. As shown in Table S3, the correlation between these two measurements was - for most antigens tested - quite similar to the correlation between percentage of positive cells and nCounter values. But for several antigens (e.g.: HLA, CD11b) the correlation coefficients were lower, probably relating to the fact that antibodies coupled to different fluorochromes were used in different patient samples for the detection of these antigens.

### Analysis of Leukemia Patients

For several antigens we then analyzed the correlation between % of blast cells in a given sample and nCounter signals. As shown in Figure 2B, samples with blasts, which did not express or only partially expressed CD34, were clearly distinct from samples with CD34 positive blasts. In the case of CD19, all samples of B-ALL showed values for CD19, which were clearly above the values for normal bone marrow, as were values for CLL or for the samples with blasts of mixed phenotype acute leukemia (MPAL).

To analyze in more detail the sensitivity of the nCounter method we performed a spiking experiment, in which we diluted a leukemia sample in a normal bone marrow sample (Table S4). Depending on the background mRNA counts for a given antigen present in the normal bone marrow sample, the nCounter was able to discriminate a diluted sample with as few as 3% blasts from the normal sample (e.g.: CD19, CD22, CD34). This high sensitivity was restricted to antigens with low background mRNA counts in normal samples; for other antigens with high background mRNA counts the sensitivity was much less (e.g. for CD58 only samples with >50% blasts could be discriminated from normal samples). The sensitivity thresholds for CD34, CD19, CD7, and CD13 can also be inferred from Figure 2.

**Figure 3:**
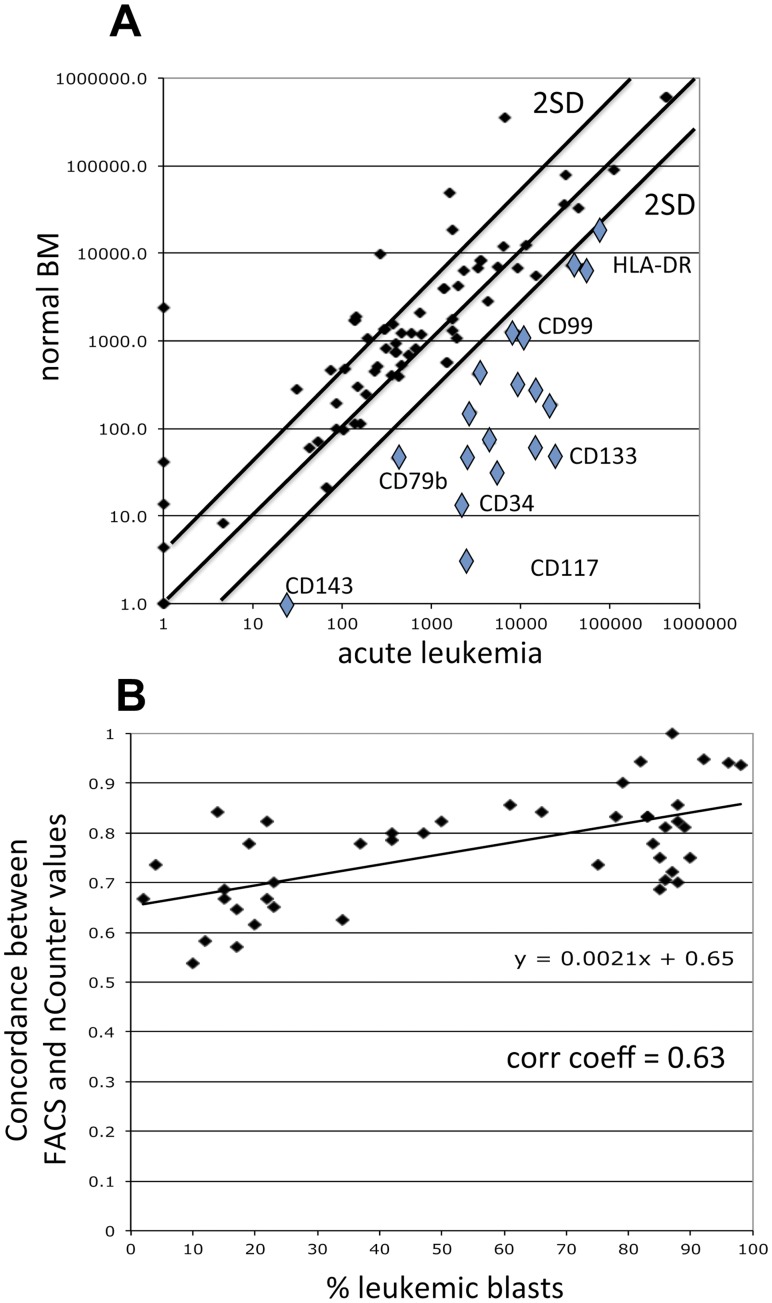
Comparative analysis of leukemia patients. **A:** Comparison of nCounter values from normal BM ( =  the mean of the values from eleven normal BM samples) with a sample from a patient with acute myeloid leukemia. Values in the acute leukemia sample, which were above the normal BM mean +2SD, were considered positive (light rectangles) and the corresponding mRNA expressed by the leukemic blasts. **B:** Correlation between % of blasts present in a leukemic sample and concordance between flow cytometry and nCounter results in forty-six patients.

For each leukemia patient the protein phenotype of the malignant blast population was determined using flow cytometry and compared to the mRNA phenotype obtained with the nCounter analysis. Two different cut-offs were used in order to consider a signal from the nCounter as “positive”: (A) the ratio of the signal in the leukemia sample/mean of the signal in the normal BM or peripheral blood sample, respectively, had to be ≥2 and, (B) the signal in the sample had to be higher than the mean of the normal BM or peripheral blood sample value +2 SD (Figure 3A). Signals fulfilling both criteria were interpreted as proof for the presence of the corresponding mRNA in the blast population.

Comparing results from flow cytometry and nCounter analyses showed that, depending on the leukemia sample analyzed, the measurements by the two methods corresponded in 44–100% of the antigens studied (mean = 76%; Table S5). Table 2 shows the correlation coefficient expressed separately for each antigen studied. When the concordance between the two methods is expressed for all antigens studied depending on the % of blasts present in the sample, a mean correlation coefficient of 0.63 was found (Figure 3B).

**Table 2:** Analysis of leukemic blasts: correlation between results from flow cytometry and nCounter analysis for twenty-two different antigens, each studied in forty-five leukemia patients.

Antigens	corr coeff
CD19	0.98
CD34	0.98
CD16	0.97
CD10	0.91
CD20	0.91
CD14	0.90
TDT	0.90
CD133	0.89
CD117	0.88
CD7	0.85
CD3	0.80
CD38	0.80
CD4	0.79
HLA-DR	0.79
MPO	0.78
CD11b	0.76
CD56	0.73
CD123	0.73
CD36	0.63
CD15	0.59
CD33	0.40
CD13	0.26

corr coeff >0.372 are statistically significant with p<0.01.

**Figure 4:**
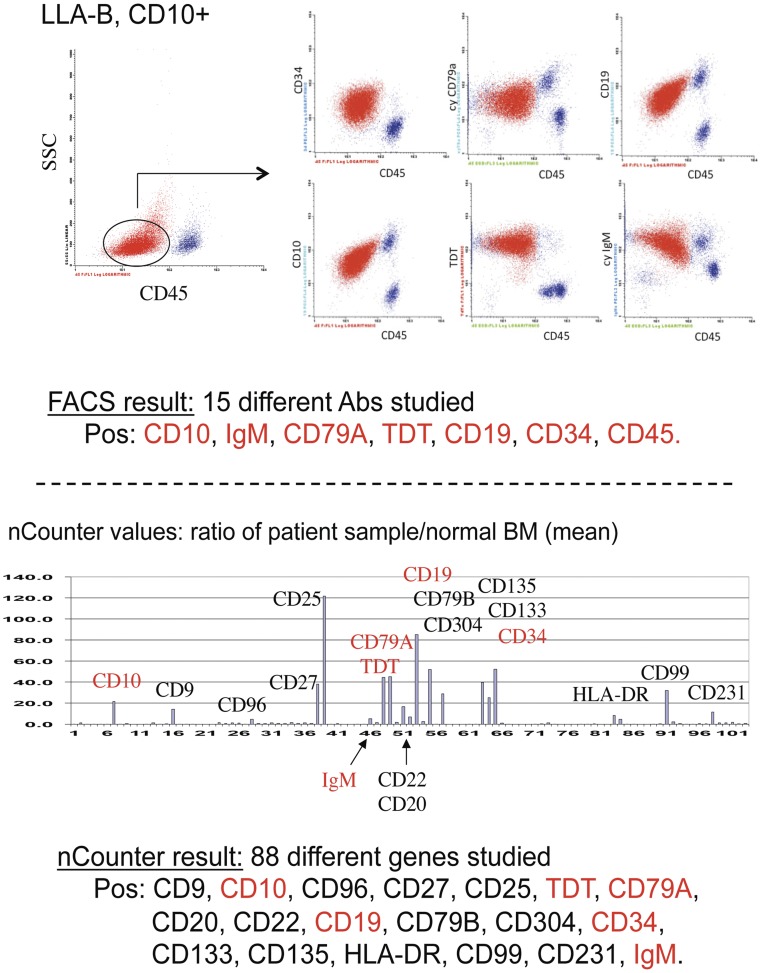
A typical case of B-ALL analyzed in parallel by flow cytometry and by the nCounter technique. Flow cytometry shows the blast population being CD34pos, CD19pos, cy CD79a pos, cy IgM pos, TDT pos, CD10 pos and CD45 pos. In red: antigens found positive with both methods.

A typical example of the type of analysis we obtained by our analysis is shown in Figure 4 for a case of B-ALL with 87% blasts. Of fifteen antigens studied by flow cytometry, seven were expressed by the blasts. With the nCounter we studied eighty-eight antigens and found RNAs of nineteen of them expressed by the blasts, including six out of the seven antigens found to be positive by flow cytometry ( =  concordance of 86%).

Discrepancies were observed in the following situations (Table S5):

- Flow cytometry results showed no protein expression on the blast cell surface, whereas the results from the nCounter were clearly positive (25% of discordant cases). This situation was encountered essentially for CD15 and CD56. Most probably, in these cases translation from mRNA to protein in the malignant blasts was defective and led to absence of the protein on the cell surface. Alternatively, the antibodies used for flow cytometry were not optimal.
- Flow cytometry results showed positive blasts, whereas the results from the nCounter were negative (75% of discordant cases). This situation occurred essentially for the myeloid genes CD13 and CD33 with correlation coefficients of 0.26 and 0.40, respectively (Table 2), and to a lesser extent for MPO, CD11b, and CD36. In fact, as the majority of the cells in normal PB and BM already express myeloid genes, it becomes impossible to distinguish samples with low or medium blast populations expressing myeloid genes from normal samples only on the basis of total mRNA quantity. Alternatively, leukemic blasts may have expressed only low amounts of a certain mRNA, which made a distinction from normal samples impossible.

### Analysis of Samples from other Swiss Centers

PB and/or BM samples from forty-three leukemia patients (Table S2) were analyzed by flow cytometry in six other Swiss centers (Aarau, Basel, Bellinzona, Bern, Lausanne, Sion), and leftover material was sent to Geneva where it was subsequently studied for mRNA expression by the nCounter technique. Two cases had to be discarded because of degraded material. Comparing results from flow cytometry and nCounter analyses for the remaining forty-one samples showed that, similarly to samples from Geneva, the two methods corresponded in 52–100% of the antigens studied (mean = 77%). Correlations were superior in ALL samples compared to AML samples, with mean correlations of 88% and 72%, respectively (Table S6).

The results of the nCounter analysis of these samples, as well as of the 45 cases from Geneva, and normal BM and PB samples are shown in table S7 and as a heatmap in figure S1.

## Discussion

Analyzing quantitatively and in parallel the expression of hundreds of different mRNAs of a given biological sample results in a wealth of information about the type of cells present, their functional status, as well as their malignant or normal nature.

In the present study we compared the expression of mRNAs coding for cell surface antigens with the expression of the corresponding proteins in hematological samples from peripheral blood and from bone marrow. We used the well-established flow cytometric technique for surface protein detection and the newly developed Nanostring technology for the mRNA determinations. Our results show excellent correlation coefficients for most of the antigens studied, thus demonstrating the comparability of both methodologically different approaches (Table 1).

Various factors might influence the degree of concordance, either at the level of mRNA detection (i.e.: number of mRNA molecules/cell for a given antigen as well as mRNA stability, amount of RNAse present in samples), or at the level of flow cytometry (e.g. quality of antibodies used, leading sometimes to nonspecific binding and overestimation of results). As our comparisons were performed on heterogeneous samples corresponding to mixtures of normal and/or malignant cell populations, differences in pre- (e.g. splice variants, mutations) or post-translational modifications between these cells might in some cases also explain weaker correlations between protein and mRNA expressions.

For most of the antigens tested comparing percentage of positive cells to nCounter values resulted in slightly higher correlation coefficients than comparing MFI values (Table S3). This reflects the greater variability in MFI measurements, which are due to the technical particularities of the flow cytometric analysis (e.g.: different antibody clones and fluorochromes result in different MFI values for the same antigen).

Our results are in line with previous similar studies, which have already investigated mRNA/protein correlations. Kern et al used gene expression profiles from standard gene expression profiling techniques (Affymetrix microarrays) and compared those to results from flow cytometry [11]. In a total of 21581 comparisons performed on thirty-six antigens, they found correlation coefficients ranging from 0.17 to 0.81. These coefficients were significantly lower than the ones obtained in our study (0.40 to 0.90). This very likely reflects the overall greater precision in RNA quantification by the nCounter exploited in our study as compared to the microarrays technologies used in the other studies (Table S8).

The emphasis of our study was on evaluating the potential of the nCounter technology for its application in acute leukemia diagnosis. Whereas in flow cytometry diagnosis is based on a multi-parameter analysis at the single cell level, the nCounter technology measures gene expression from the total unsorted cell population of a sample. It can therefore not substitute flow cytometry.

In order to be informative the signals from the nCounter have to clearly distinguish pathologic from normal samples. Using two different algorithms we defined nCounter signals as positive and as expressed by the blast population in a given sample. Applying this strategy, we found concordance in 44–100% of the antigens studied, depending on the number of blasts present in a sample as well as on the type of leukemia. Concordance up to 100% was seen mainly in B- and T-ALL samples with high blast numbers, as well as in some rare forms of leukemia (e.g. AML M7, leukemic blastic plasmacytoid dendritic cell neoplasm), corresponding to situations in which the normal counterpart of the malignant blast population constituted only a minor fraction of the total bone marrow or peripheral blood cell population (Tables S5 and S6). In these cases samples with ≥10% of blasts were clearly distinguished from normal cases with <2% blast cells (Figure 2A and 2B) and concordance between protein and mRNA expressions was high. For leukemias with aberrant myeloid cells concordance was lower, in particular for the myeloid antigens CD13 and CD33 (Table 2).

In order to show the potential utility of the nCounter technology for multi-center studies, patients were tested first by flow cytometry in six different Swiss centers and leftover samples were then centrally analyzed in Geneva with the nCounter. The concordance between protein and mRNA expressions (mean = 75%) was practically as high as the concordance for the Geneva samples (mean = 76%). We expect improvement in concordance if special pre-analytical precautions for the send-out of the samples are taken (e.g. PAX Gene tubes to inhibit mRNA degradation), or if flow cytometry procedures are standardized. From a practical point of view, the ease-of-use of this technology, the short turnaround time of typically less than two days, as well as the possibility to use crude cell lysates for the mRNA profiling offers immense advantages compared to previously used approaches of mRNA analysis [12].

In summary, our study shows a high correlation between the expression of surface proteins and of mRNA in peripheral blood or bone marrow samples, using the well-established flow cytometry and the newly developed nCounter technology, respectively. The “CD antigen profile” obtained by the nCounter is easily extendable to several hundreds of antigens, which can be tested in one single assay and can thus supplement for antigens for which routine flow cytometry laboratories do not have the necessary antibodies available. Furthermore, extension of the antigen profile, potentially picking up aberrant expression of several antigens simultaneously, could compensate in some cases for the lack of a gating strategy by flow cytometry. In a similar way a large antigen profile could become particularly useful for the determination of the exact phenotype of mixed lineage acute leukemias (MPAL) and for the diagnosis of rare types of acute leukemias.

## Materials and Methods

### Samples

Fresh peripheral blood (PB) and bone marrow (BM) samples were obtained from patients of the Geneva Hematology Service in accordance with institutional guidelines and after approval of the ethical committee of the Geneva University Hospital (Table S2). Written informed consent was obtained from all patients. Normal blood samples were obtained from blood donors of the regional blood transfusion center. CD14+ monocytes, CD8+ T cells, CD56+ NK cells, CD19+ B cells, neutrophils, and eosinophils were isolated from these samples using Enrichment and Selection Kits from Stemcell Technologies, according to manufacturer’s instructions. CD4+ T cells were isolated from the monocytes-depleted fraction (obtained during the monocytes isolation) using the EasySep® Human CD4 Positive Selection Kit (Stemcell Technologies). Purity of the isolated cell populations was verified by flow cytometry with specific antibodies and was >80% in all cases (data not shown).

From each sample (PB, BM, or enriched cell populations) 10^6^ cells were lysed in RNA lysis buffer (Qiagen, Venlo, Netherlands) and stored at −80°C for further analysis.

### Samples from other Centers

From other Swiss Centers (Aarau, Basel, Bellinzona, Bern, Lausanne, Sion) leftover samples from the flow cytometric analysis of forty-three leukemic patients were obtained after approval of the respective ethical committees (ethical committee of Kantonsspital Aarau, ethical committee of Inselspital Bern, ethical committee of University Hospital Lausanne, ethical committee of Institut Central des Hôpitaux Valaisans, ethical Committee of Istituto Oncologico della Svizzera Italiana) and were sent to Geneva by mail, which took one to two days (Table S2). Written informed consent was obtained from all patients. At arrival, the samples were centrifuged over a Ficoll-Gradient to remove apoptotic and dead cells, and the number of leukemic blasts was determined by flow cytometry using a standard 5-color tube (CD7/CD19/CD33/CD34/CD45). The samples were then stored for further analysis at −80°C.

### Flow Cytometry

Flow cytometric analysis was performed with monoclonal 5-color antibody panels (Table S9A). In short, samples were incubated for 15 min at RT with the corresponding antibodies, a lysis solution (Optilyse®C, Beckman Coulter, Miami, FL) was added for 10 min to lyse red bood cells, the cells were washed twice, and than analyzed on a FC500 flow cytometer (Beckman Coulter). White blood cell populations were analyzed after gating out debris, cell aggregates, dead cells, platelets and red blood cells on FSC/SSC and CD45/SSC dotblots. Analysis of list-mode files was performed using the FACSExpress software (De Novo Software; Los Angeles; CA) with the analysis gate set in the CD45/side-scatter (SSC) - plot to include all nucleated cells (i.e.: lymphocytes, monocytes, myeloid cells and blasts). The percentage of different cell-subpopulations was determined using anti-CD4 and anti-CD8 antibodies for T lymphocytes, anti-CD19 for B lymphocytes, anti-CD14 for monocytes, and the CD45/SSC pattern for myeloid cells. The percentage of blasts was determined, using either a combination of CD45 dim/SSC low pattern and anti-CD34, anti-CD117 or anti-CD133 antibodies for myeloid blasts or anti-CD19 or anti-CD7 antibodies for B and T lymphoid blasts, respectively. For each antigen analyzed the percentage of positive cells was determined in relation to isotype controls, which were set arbitrarily at 1% positivity. The mean fluorescent intensity (MFI) for each antigen was determined as the ratio of the antigen values and the isotype control value.

### mRNA Analysis

The RNA from the equivalent of 10^4^ cells (corresponding to 100–300 ng total RNA) was used for the analysis with the nCounter system, according to the manufacturer’s protocol (Nanostring ® Technologies, Seattle, WA, USA). In brief, 4 ul of cell lysate (in RLT buffer, according to Qiagen recommendations) was hybridized at 65°C over night with the Nanostring Codeset.

Probes for the analysis of ninety-six different antigens (Table S9B) were synthesized by Nanostring technologies, including probes for seventy-six surface antigens, twelve intracellular antigens, and eight normalization genes (Table S10). After probe hybridizations and Nanostring nCounter digital reading, counts for each RNA species were extracted, analyzed using a homemade Excel macro, and then expressed as counts (molecules of mRNA/sample) [8]. The nCounter CodeSet contained two types of built-in controls: positive controls (spiked RNA at various concentrations to assess the overall assay performance), and negative controls (alien probes for background calculation). Data handling and analysis was performed as described: background correction consisted of the subtraction of the negative control average plus two SD from the original counts. To select adequate normalization genes from a series of candidates included in the CodeSet, their relative stability was evaluated using geNorm-method [13]. For the final normalization of the sample values the geometric mean of the counts obtained for the selected normalization genes was calculated and used as normalization factor.

In preliminary experiments one sample from normal PB, one from normal BM, and one tumor sample were analyzed in triplicates in several runs on the nCounter machine (Figure S2). The ratio SD/mean for each value was <0.5 for values between 50 and 10^6^, and >0.5 to 1.1 for values between 1 and 50 for all three triplicates. Similar low intra-assay variations have already been observed by others [8]. We decided for the rest of our study to perform each analysis for each sample only once.

The dynamic range and linearity of the assay were tested in a dilution experiment (Figure S3).

Over a range of 2.5–3 log highly linear results were obtained, in agreement with the results reported in the original description of the technique [7].

### Statistics

nCounter and flow cytometry results from a total of twenty-two antigens were compared in eighty-six leukemia cases (forty-five cases from Geneva and forty-one cases from other Swiss centers), applying Pearson’s correlation.

## Supporting Information

Figure S1 **Heatmap for nCounter values (mRNA counts) obtained for normal and leukemic samples, according to table S7.** Hierarchical supervised 2-way clustering was performed with an Euclidean distance on the log2 of the signal intensity. The values were centered around zero and color-coded ranging from −6 to 6, according to the log2 value.
(TIF)

Figure S2 **Technical reproducibility of the nCounter assay.** A tumor sample, a sample from a normal BM and a normal peripheral blood sample were analyzed each in three different runs with the nCounter. The graphs depict the comparison between the different runs (log values), as well as the corresponding correlation coefficients.
(TIF)

Figure S3 **Linearity of the nCounter assay.** A normal bone marrow sample was diluted 1/3,1/9 and 1/27 and analyzed with the nCounter. Shown are the results obtained for the twenty-five most expressed antigens. Trendlines for two antigens are depicted.
(TIF)

Table S1 **“CD mRNA phenotype” of normal peripheral blood and bone marrow.** Columns correspond to mean nCounter values from peripheral blood (5) and bone marrow (11) samples, ordered according to their expression in different cell populations. SD  =  standard deviation.
(DOCX)

Table S2 **Sample characteristics.** Samples from Geneva and from other Swiss centers (Aarau, Basel, Bellinzona, Bern, Lausanne, Sion) were used for this study. *one plasmacytoid dendritic cell leukemia (leukemic BPDC according to WHO 2008); one MDS (RCMD); one juvenile MM leukemia. Three samples (1 from Geneva and 2 from other centers) were degraded and not used for nCounter analysis.
(DOC)

Table S3 **Correlation coefficients (Pearson) between flow cytometry and nCounter.** (A) Percentage of positive cells and values from the nCounter analysis. (B) MFI values and values from the nCounter analysis.
(DOC)

Table S4 **A BM sample from a patient with B-ALL containing 98% blasts was analyzed with the nCounter.** Samples were run undiluted, and diluted 1/2, 1/4, 1/8, 1/16 and 1/32 in normal BM. Genes, which were expressed by the leukemic blasts, are highlighted in gray, as well as the dilutions at which a distinction between leukemic and normal sample was possible ( =  sensitivity of the assay for the detection of this gene).
(XLSX)

Table S5 **CD protein and mRNA phenotype analysis of forty-five leukemia patients with flow cytometry and nCounter technology.** The forty-five samples were ordered according to the % of concordance (column E) between results from nCounter technology and flow cytometry. Sample No 36 out of the original forty-six samples was not analysed (degraded material).
(DOCX)

Table S6 **Concordance between Flow Cytometry and nCounter values for forty-one different leukemia samples obtained from other Swiss Centers.** The forty-one samples were ordered according to the % of concordance between results from nCounter technology and flow cytometry. Two of the original forty-three samples were not analysed (degraded material).
(XLSX)

Table S7 **nCounter values (mRNA counts) for normal and leukemic samples Samples (nBM, normal bone marrow; nPB, normal peripheral blood; AML, LLA-B, LLA-T) are ordered in columns and the genes tested in lines.** 88 genes were tested. For several genes values were not obtained for all samples due to a restricted codeset used for the analysis (ND; not done).
(XLS)

Table S8 **Comparison of the data from the study by Kern et al. with our own data.** Correlation coefficients (Spearman) are shown for twenty-two antigens. Legend: in bold are correlation coefficients, which are higher in our study compared to Kern et al.’s study (ND; not done).
(DOC)

Table S9 **Reagents for flow cytometry and nCounter analyses.** A: Antibodies used for flow cytometric analysis. B: Probes used in the nCounter analysis.
(DOC)

Table S10 **Target sequences for probes used in the nCounter analysis.**
(XLSX)
