# Supplementary figures and images for: Using Digital RNA Counting and Flow Cytometry to Compare mRNA with Protein Expression in Acute Leukemias
Source: PLoS One. 2012 Nov 9;7(11):e49010. doi: 10.1371/journal.pone.0049010 (PMC3494663; doi:10.1371/journal.pone.0049010)

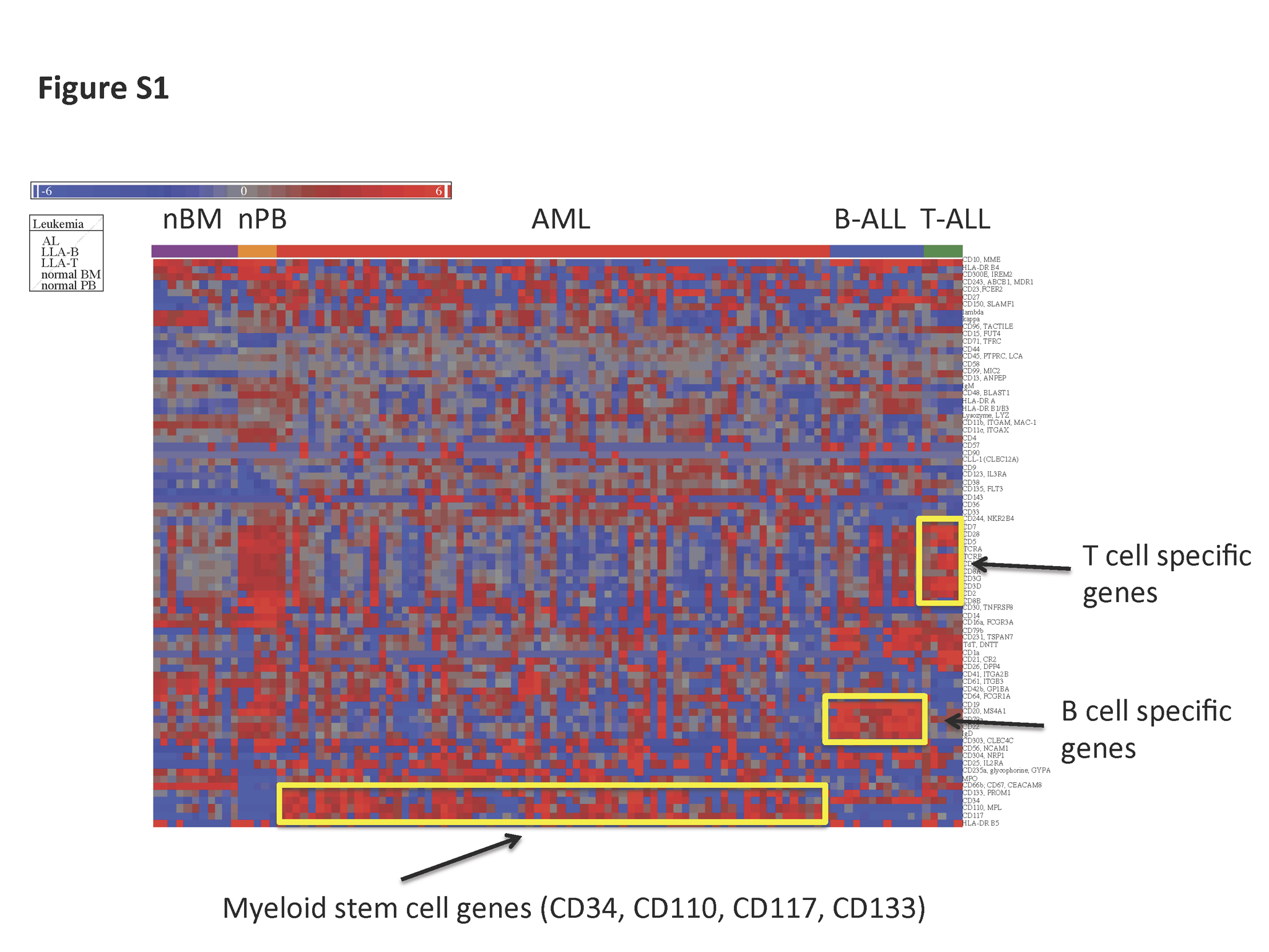

Supplement: Figure S1 — Heatmap for nCounter values (mRNA counts) obtained for normal and leukemic samples, according to table S7. Hierarchical supervised 2-way clustering was performed with an Euclidean distance on the log2 of the signal intensity. The values were centered around zero and color-coded ranging from −6 to 6, according to the log2 value. (TIF) [file pone.0049010.s001.tif]

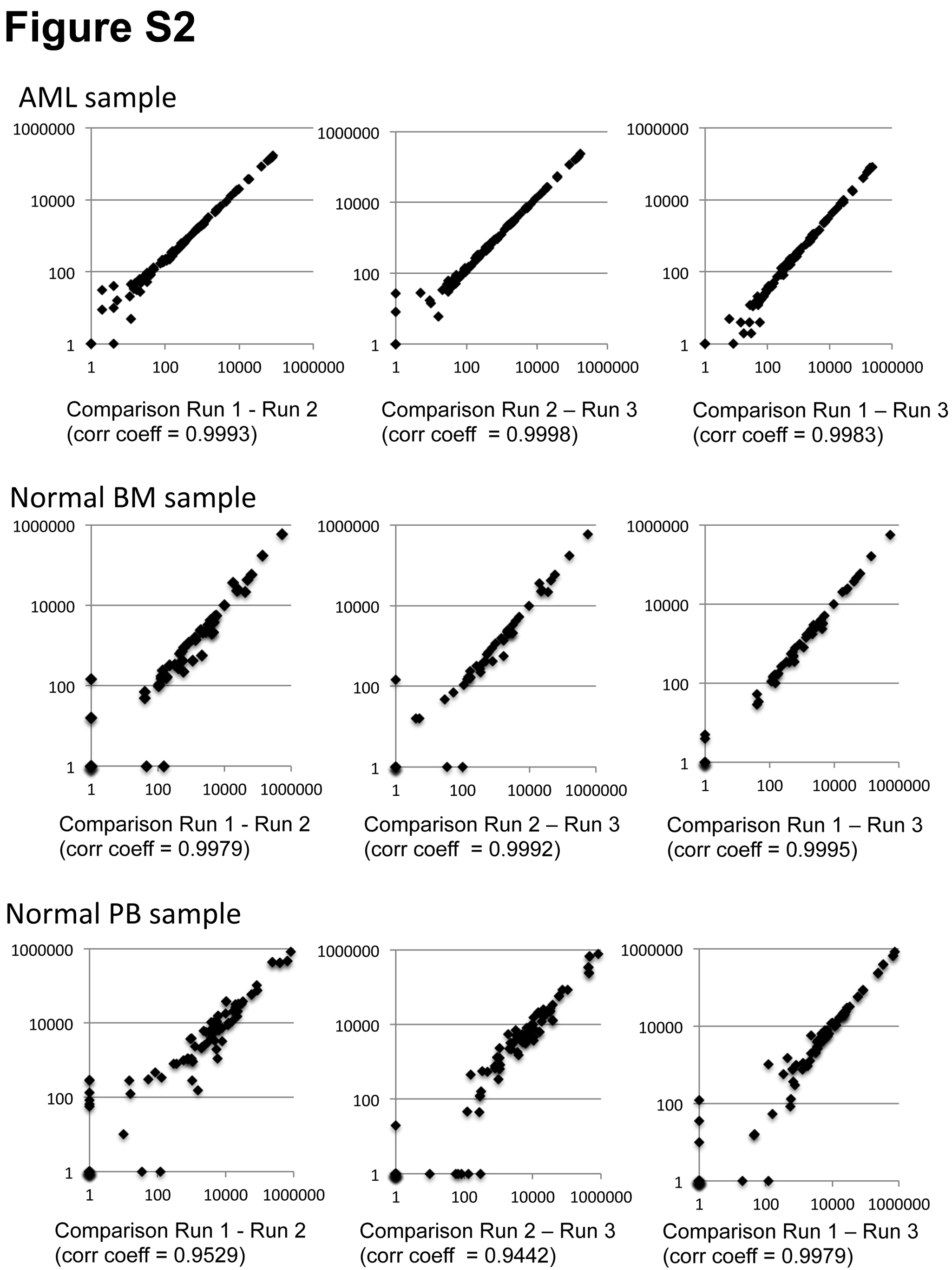

Supplement: Figure S2 — Technical reproducibility of the nCounter assay. A tumor sample, a sample from a normal BM and a normal peripheral blood sample were analyzed each in three different runs with the nCounter. The graphs depict the comparison between the different runs (log values), as well as the corresponding correlation coefficients. (TIF) [file pone.0049010.s002.tif]

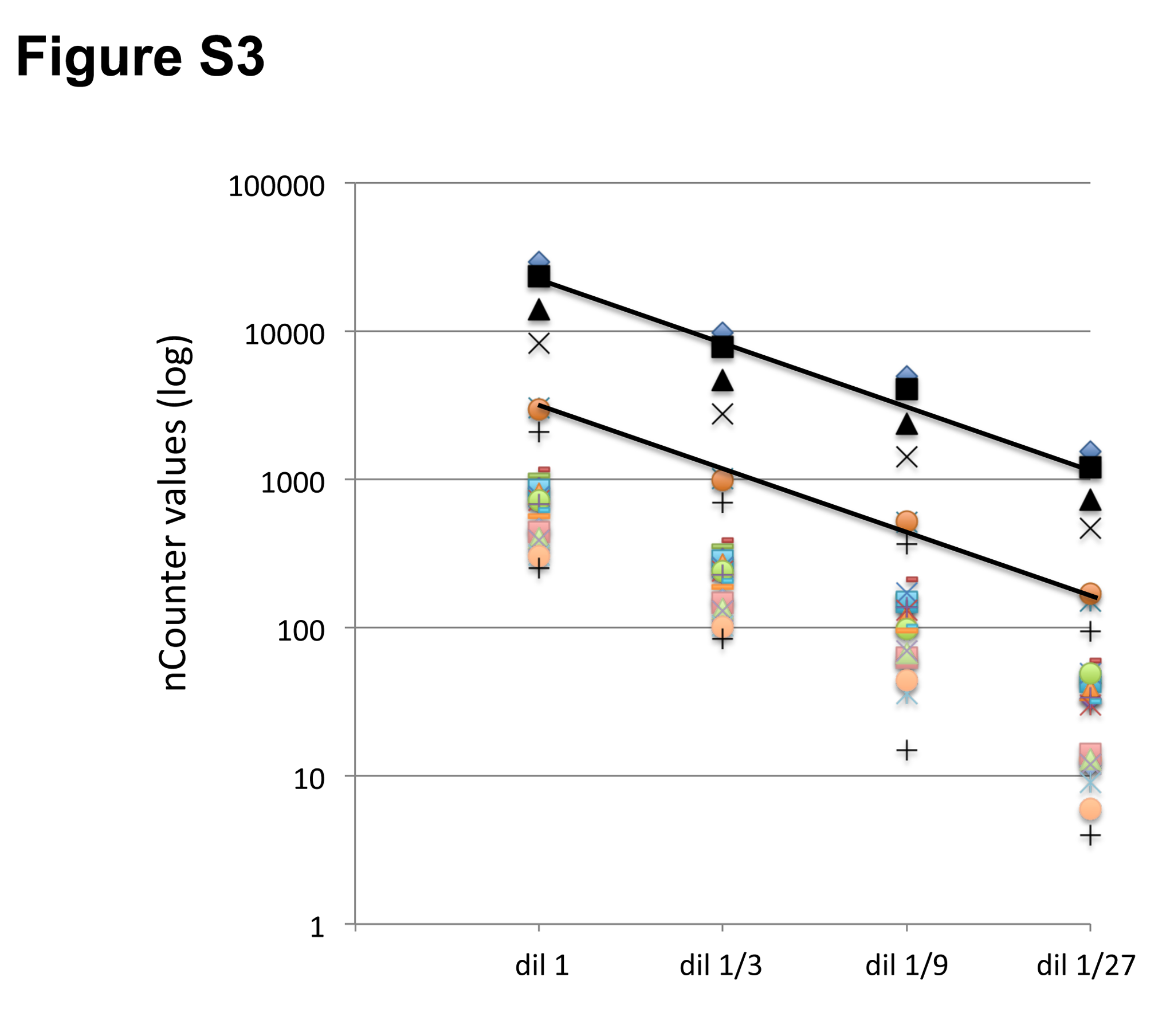

Supplement: Figure S3 — Linearity of the nCounter assay. A normal bone marrow sample was diluted 1/3,1/9 and 1/27 and analyzed with the nCounter. Shown are the results obtained for the twenty-five most expressed antigens. Trendlines for two antigens are depicted. (TIF) [file pone.0049010.s003.tif]
