# Supplementary material for: Using Digital RNA Counting and Flow Cytometry to Compare mRNA with Protein Expression in Acute Leukemias
Source: PLoS One. 2012 Nov 9;7(11):e49010. doi: 10.1371/journal.pone.0049010 (PMC3494663; doi:10.1371/journal.pone.0049010)
Supplement: Table S1 — “CD mRNA phenotype” of normal peripheral blood and bone marrow. Columns correspond to mean nCounter values from peripheral blood (5) and bone marrow (11) samples, ordered according to their expression in different cell populations. SD = standard deviation. (DOCX) [file pone.0049010.s004.docx]

**Table S1:** CD mRNA phenotype of normal peripheral blood and bone marrow
