# Supplementary material for: Using Digital RNA Counting and Flow Cytometry to Compare mRNA with Protein Expression in Acute Leukemias
Source: PLoS One. 2012 Nov 9;7(11):e49010. doi: 10.1371/journal.pone.0049010 (PMC3494663; doi:10.1371/journal.pone.0049010)
Supplement: Table S2 — Sample characteristics. Samples from Geneva and from other Swiss centers (Aarau, Basel, Bellinzona, Bern, Lausanne, Sion) were used for this study. *one plasmacytoid dendritic cell leukemia (leukemic BPDC according to WHO 2008); one MDS (RCMD); one juvenile MM leukemia. Three samples (1 from Geneva and 2 from other centers) were degraded and not used for nCounter analysis. (DOC) [file pone.0049010.s005.doc]

**Table S2:** Sample characteristics

#

|  | Geneva | Other Swiss Centers |
| --- | --- | --- |
| Normal bone marrow | 11 |  |
| AML | **36** | **31** |
| MPAL | 1 | 4 |
| AML M0 | 3 | 2 |
| AML M1/M2 | 23 | 12 |
| AML M3 | 3 | 4 |
| AML M4/M5 | 4 | 9 |
| AML M6 | 1 | 0 |
| AML M7 | 1 | 0 |
|  |  |  |
| B-ALL | **8** | **6** |
| T-ALL | **2** | **3** |
|  |  |  |
| CLL | 3 |  |
| CML | 2 |  |
|  |  |  |
| other hematologic malignancies* |  | 3 |
