# Supplementary material for: Using Digital RNA Counting and Flow Cytometry to Compare mRNA with Protein Expression in Acute Leukemias
Source: PLoS One. 2012 Nov 9;7(11):e49010. doi: 10.1371/journal.pone.0049010 (PMC3494663; doi:10.1371/journal.pone.0049010)
Supplement: Table S3 — Correlation coefficients (Pearson) between flow cytometry and nCounter. (A) Percentage of positive cells and values from the nCounter analysis. (B) MFI values and values from the nCounter analysis. (DOC) [file pone.0049010.s006.doc]

**Table S3:** Correlation coefficients (Pearson) between flow cytometry and nCounter.

(A) % of positive cells (flow cytometry) and values from the nCounter analysis (mRNA counts).

(B) MFI values (flow cytometry) and values from the nCounter analysis (mRNA counts).

| **Correlation**  **Coefficient** | **A** | **B** |
| --- | --- | --- |
| **Antigens** | **% pos cells** | **MFI** |
| CD34 | 0.82 | 0.63 |
| HLA | 0.76 | 0.37 |
| CD117 | 0.75 | 0.57 |
| CD11b | 0.78 | 0.52 |
| CD7 | 0.76 | 0.96 |
| CD10 | 0.81 | 0.84 |
| CD133 | 0.97 | 0.98 |
| CD19 | 0.83 | 0.66 |
| CD20 | 0.89 | 0.64 |
| CD56 | 0.80 | 0.86 |
| CD14 | 0.56 | 0.56 |
| CD3E | 0.94 | 0.80 |
| MPO | 0.53 | 0.36 |
| CD13 | 0.61 | 0.47 |
| CD4 | 0.68 | 0.55 |
| CD16 | 0.74 | 0.68 |
| CD33 | 0.49 | 0.57 |
| CD36 | 0.57 | 0.49 |
| TDT | 0.89 | 0.83 |
| CD38 | 0.40 | 0.93 |
| CD123 | 0.53 | 0.98 |
| CD15 | 0.41 | 0.31 |
