# Supplementary material for: Using Digital RNA Counting and Flow Cytometry to Compare mRNA with Protein Expression in Acute Leukemias
Source: PLoS One. 2012 Nov 9;7(11):e49010. doi: 10.1371/journal.pone.0049010 (PMC3494663; doi:10.1371/journal.pone.0049010)
Supplement: Table S5 — CD protein and mRNA phenotype analysis of forty-five leukemia patients with flow cytometry and nCounter technology. The forty-five samples were ordered according to the % of concordance (column E) between results from nCounter technology and flow cytometry. Sample No 36 out of the original forty-six samples was not analysed (degraded material). (DOCX) [file pone.0049010.s008.docx]

**Table S5:** Leukemia patients (Geneva)

Comparison between data from nCounter and flow cytometry

|  | |  |  |  |  |  |  |  |
| --- | --- | --- | --- | --- | --- | --- | --- | --- |
|  | | | | |  |  |  |  |
|  |  |  |  |  |  |  |  |  |
|  |  |  |  |  | **Discrepancies** | |  |  |
| **Diagnosis** | blasts | Total antigens | Concordant | **concordance** | nCounter pos; | Antigens | nCounter neg; | Antigens |
|  | % | studied | results | **%** | Flow neg |  | Flow pos |  |
|  |  |  |  |  |  |  |  |  |
| LLA-B | 87 | 9 | 9 | 100% | 0 |  | 0 |  |
| LMA M1/M2 | 92 | 19 | 18 | 95% | 0 |  | 1 | CD33 |
| LLA-B | 82 | 18 | 17 | 94% | 1 | CD20 | 0 |  |
| LLA-B | 96 | 17 | 16 | 94% | 1 | CD15 | 0 |  |
| LLA-B | 98 | 16 | 15 | 94% | 1 | CD117 | 0 |  |
| LMA M1/M2 | 79 | 10 | 9 | 90% | 0 |  | 1 | CD13 |
| LMA M7 | 88 | 14 | 12 | 86% | 1 | CD15 | 1 | CD4 |
| LMA M1/M2 | 61 | 14 | 12 | 86% | 0 |  | 2 | CD13, CD33 |
| LMA M1/M2 | 66 | 19 | 16 | 84% | 0 |  | 3 | CD123, CD13, CD33 |
| LMA M1/M2 | 14 | 19 | 16 | 84% | 0 |  | 3 | CD13, CD33, CD15 |
| LMA M3 | 83 | 18 | 15 | 83% | 1 | CD4, | 2 | CD36, CD13 |
| LMA M3 | 83 | 18 | 15 | 83% | 1 | CD56, | 2 | CD36, CD13 |
| LMA M4/M5 | 78 | 12 | 10 | 83% | 1 | CD117 | 1 | CD13 |
| LMA M0 | 50 | 17 | 14 | 82% | 1 | CD14 | 2 | CD7, CD33 |
| LMA M1/M2 | 88 | 17 | 14 | 82% | 0 |  | 3 | CD33, CD13, MPO |
| LMA M1/M2 | 22 | 17 | 14 | 82% | 0 |  | 3 | CD36, HLA, CD33 |
| LMA M1/M2 | 89 | 16 | 13 | 81% | 1 | CD56 | 2 | CD4, CD13 |
| LMA M4/M5 | 86 | 16 | 13 | 81% | 1 | CD4, | 2 | CD36, CD11b |
| LMA M0 | 47 | 15 | 12 | 80% | 2 | CD19, CD14 | 1 | CD13 |
| MPAL | 42 | 10 | 8 | 80% | 0 |  | 2 | CD33, CD13 |
| LMA M1/M2 | 42 | 14 | 11 | 79% | 1 | CD56 | 2 | CD13, HLA |
| LMA M1/M2 | 19 | 9 | 7 | 78% | 0 |  | 2 | CD13, CD33 |
| LLA-T | 37 | 18 | 14 | 78% | 1 | CD3 | 3 | TDT, CD11b, CD33 |
| LLA-T | 84 | 9 | 7 | 78% | 1 | CD3 | 1 | CD33 |
| LMA M1/M2 | 85 | 20 | 15 | 75% | 2 | CD133, CD15 | 3 | CD10, CD11b, CD13 |
| LMA M4/M5 | 90 | 16 | 12 | 75% | 1 | CD15 | 3 | CD11b, CD13, CD33 |
| LMA M1/M2 | 4 | 19 | 14 | 74% | 2 | CD4, CD15 | 3 | HLA, CD13, CD33 |
| LMA M1/M2 | 75 | 19 | 14 | 74% | 0 |  | 5 | CD36, CD38, CD11b, CD13, CD33 |
| LMA M4/M5 | 87 | 18 | 13 | 72% | 1 | CD117 | 4 | MPO , CD11b, CD13, CD14 |
| LMA M1/M2 | 86 | 17 | 12 | 71% | 1 | TDT | 4 | MPO, CD15, CD13, CD33 |
| LMA M3 | 88 | 10 | 7 | 70% | 2 | CD15, CD34 | 1 | CD13 |
| LLA-B | 23 | 10 | 7 | 70% | 1 | CD7 | 2 | CD10, CD33 |
| LMA M0 | 15 | 16 | 11 | 69% | 2 | CD7, CD56 | 3 | CD33. CD13, MPO |
| LMA M1/M2 | 85 | 16 | 11 | 69% | 1 | CD15 | 4 | MPO, CD11b, HLA, CD13 |
| LMA M1/M2 | 22 | 9 | 6 | 67% | 1 | CD56 | 2 | CD33, CD13 |
| LLA-B | 15 | 6 | 4 | 67% | 0 |  | 2 | CD38, CD10 |
| LMA M1/M2 | 23 | 20 | 13 | 65% | 1 | CD15, | 6 | CD123, CD133, MPO, HLA, CD13, CD33 |
| LMA M1/M2 | 17 | 17 | 11 | 65% | 2 | CD36, CD56 | 4 | TDT, HLA, CD13, CD33 |
| LMA M1/M2 | 34 | 8 | 5 | 63% | 0 |  | 3 | CD123, CD33, CD7 |
| LMA M1/M2 | 20 | 13 | 8 | 62% | 2 | MPO, CD15 | 3 | HLA, CD13, CD33 |
| LMA M6 | 12 | 12 | 7 | 58% | 0 |  | 5 | CD4, CD15, CD13, CD33, CD7 |
| LMA M1/M2 | 17 | 7 | 4 | 57% | 0 |  | 3 | CD33, CD13, CD16a |
| LMA M1/M2 | 10 | 13 | 7 | 54% | 1 | CD56 | 5 | CD36, CD117, CD33, CD13, CD7 |
| LLA-B | 7 | 9 | 4 | 44% | 2 | CD5, MPO | 3 | CD10, CD19, CD33 |
| LMA M1/M2 | 46 | 7 | 3 | 43% | 1 | CD14 | 3 | CD7, CD33, CD34 |
|  |  |  |  |  |  |  |  |  |
| **Sum** |  | **643** | **495** | **MEAN =76%** | **38** |  | **110** |  |
|  |  |  |  |  |  |  |  |  |
|  |  |  |  |  |  |  |  |  |
