# Supplementary material for: Using Digital RNA Counting and Flow Cytometry to Compare mRNA with Protein Expression in Acute Leukemias
Source: PLoS One. 2012 Nov 9;7(11):e49010. doi: 10.1371/journal.pone.0049010 (PMC3494663; doi:10.1371/journal.pone.0049010)
Supplement: Table S8 — Comparison of the data from the study by Kern et al. with our own data. Correlation coefficients (Spearman) are shown for twenty-two antigens. Legend: in bold are correlation coefficients, which are higher in our study compared to Kern et al.’s study (ND; not done). (DOC) [file pone.0049010.s011.doc]

**Table S8:** Comparison of the correlation coefficients (Spearman) from our study and the study by Kern et al.

| CC (Spearman) | Our study | Kern et al. |
| --- | --- | --- |
| CD34 | **0.90** | 0.81 |
| HLA | 0.88 | ND |
| CD117 | **0.87** | 0.73 |
| CD11b | **0.87** | 0.63 |
| CD7 | **0.86** | 0.65 |
| CD10 | 0.79 | ND |
| CD133 | **0.79** | 0.68 |
| CD19 | **0.77** | 0.17 |
| CD20 | 0.77 | 0.80 |
| CD56 | **0.77** | 0.40 |
| CD14 | 0.71 | 0.76 |
| CD3 | **0.71** | 0.26 |
| MPO | 0.69 | 0.72 |
| CD13 | **0.69** | 0.60 |
| CD4 | **0.69** | 0.63 |
| CD16 | 0.68 | ND |
| CD33 | 0.59 | 0.72 |
| CD36 | 0.59 | 0.66 |
| TDT | **0.53** | 0.45 |
| CD38 | **0.53** | 0.46 |
| CD123 | 0.52 | ND |
| CD15 | 0.40 | 0.49 |
