# Supplementary material for: Using Digital RNA Counting and Flow Cytometry to Compare mRNA with Protein Expression in Acute Leukemias
Source: PLoS One. 2012 Nov 9;7(11):e49010. doi: 10.1371/journal.pone.0049010 (PMC3494663; doi:10.1371/journal.pone.0049010)
Supplement: Table S9 — Reagents for flow cytometry and nCounter analyses. A: Antibodies used for flow cytometric analysis. B: Probes used in the nCounter analysis. (DOC) [file pone.0049010.s012.doc]

**Table S9:** Reagents

# Table S 9A: Antibodies used in flow cytometry

**Surface antigens (54):**

CD1a, CD2, CD3, CD4, CD5, CD7, CD8A, CD8B, CD10, CD11b, CD11c, CD13, CD14, CD15, CD16a, CD19, CD20, CD21, CD22, CD23, CD25, CD26, CD27, CD28, CD30, CD33, CD34, CD36, CD38, CD41, CD42b, CD44, CD45, CD56, CD57, CD58, CD61, CD64, CD67, CD68, CD71, CD79a, CD117, CD123, CD133, CD235a, TCRA, TCRB, kappa, lambda, IgD, IgM, HLA-DR A, HLA-DR B

**Intracellular antigens (3):**

MPO, TDT, Lysozyme

# Table S 9B: probes used for the nCounter analysis

**Surface antigens (76):**

CD1a, CD2, CD3, CD4, CD5, CD7, CD8A, CD8B, CD9, CD10, CD11b, CD11c, CD13, CD14, CD15, CD16a, CD19, CD20, CD21, CD22, CD23, CD25, CD26, CD27, CD28, CD30, CD33, CD34, CD36, CD38, CD41, CD42b, CD44, CD45, CD48, CD56, CD57, CD58, CD61, CD64, CD67, CD68, CD71, CD79a, CD79b, CD85g, CD90, CD96, CD99, CD110, CD114, CD116, CD117, CD123, CD133, CD135, CD143, CD150, CD163, CD200, CD203c, CD231, CD235a, CD243, CD244, CD300E, CD303, CD304, TCRA, TCRB, kappa, lambda, IgD, IgM, HLA-DR A, HLA-DR B

**Intracellular antigens (12):**

MPO, TDT, Elastase, Lactotransferrin, ALAS2, CA1, PRG2, Tryptase, Ki67, CLL-1, Lysozyme, TCL1A

**Normalisation genes (8):**

ACTB, B2M, GAPDH, PPIA, PGK1, G6PD, ALAS1, TBP
